# Generation of a novel transgenic rat model for tracing extracellular vesicles in body fluids

**DOI:** 10.1038/srep31172

**Published:** 2016-08-19

**Authors:** Aya Yoshimura, Masaki Kawamata, Yusuke Yoshioka, Takeshi Katsuda, Hisae Kikuchi, Yoshitaka Nagai, Naoki Adachi, Tadahiro Numakawa, Hiroshi Kunugi, Takahiro Ochiya, Yoshitaka Tamai

**Affiliations:** 1 Division of Laboratory Animals Resources, National Institute of Neuroscience, National Center of Neurology and Psychiatry (NCNP), Tokyo, Japan; 2 Department of Mental Disorder Research, National Institute of Neuroscience, NCNP, Tokyo, Japan; 3 Division of Molecular and Cellular medicine, National Cancer Center Research Institute (NCC), Japan; 4 Department of Degenerative Neurological Diseases, National Institute of Neuroscience, NCNP, Tokyo, Japan; 5 Department of Neurotherapeutics, Osaka University Graduate School of Medicine, Osaka, Japan; 6 Department of Biomedical Chemistry, School of Science and Technology, Kwansei Gakuin University, Sanda, Japan; 7 Department of Cell Modulation, Institute of Molecular Embryology and Genetics, Kumamoto University, Kumamoto, Japan

## Abstract

Extracellular vesicles (EVs) play an important role in the transfer of biomolecules between cells. To elucidate the intercellular transfer fate of EVs *in vivo*, we generated a new transgenic (Tg) rat model using green fluorescent protein (GFP)-tagged human CD63. CD63 protein is highly enriched on EV membranes via trafficking into late endosomes and is often used as an EV marker. The new Tg rat line in which human CD63-GFP is under control of the CAG promoter exhibited high expression of GFP in various body tissues. Exogenous human CD63-GFP was detected on EVs isolated from three body fluids of the Tg rats: blood serum, breast milk and amniotic fluid. *In vitro* culture allowed transfer of serum-derived CD63-GFP EVs into recipient rat embryonic fibroblasts, where the EVs localized in endocytic organelles. These results suggested that this Tg rat model should provide significant information for understanding the intercellular transfer and/or mother-child transfer of EVs *in vivo*.

Extracellular vesicles (EVs) are small membrane vesicles (40–200 nm in diameter) that are secreted from many different cell types in the body. EVs are originated from the plasma membrane and endosomes^1^. The former are vesicles called as microvesicles generated by outward budding from plasma membrane. The latter are formed by invagination and budding of early endosomes and the endosomes containing a number of vesicles inside the lumen are called multivesicular bodies (MVBs). The MVBs release vesicles called exosomes into the extracellular environment by fusion with the plasma membrane. EVs can contain lipids, proteins and RNAs, including microRNAs (miRs), and the compositions depend on the cellular origin. EVs collected from the cerebrospinal fluid (CSF) of brain-injured patients include inflammation-related proteins^2^, and EVs from the CSF of glioblastoma patients exhibits increased miR-21 levels, which is a highly overexpressed miR in glioblastoma cells^3^. These results have suggested that EVs may offer important information on pathological conditions as a novel biomarker. The EVs released into the extracellular space can be incorporated into the internal components to target cells; alternatively, the receptors and antigens (e.g., proteins, ligands) on EVs can activate cell surface receptors of target cells^4^. The ability of EVs to affect gene expression extends not only to neighbour cells but also to distant cells, because EVs circulate through the bloodstream and other body fluids^5^,^6^.

To facilitate further investigation of EV functions in cell-cell communication *in vivo*, we generated a new transgenic (Tg) rat model using EVs labelled with green fluorescent protein (GFP). Whereas most relevant studies have focused on EV functions *in vitro*, its roles *in vivo* such as EV transfer among tissues in the body of an individual or from mother to child via breast milk or placenta are still uncertain. Several studies have suggested that EVs target specific cells depending on the type of cell from which the EVs are released. Frühbeis and colleagues have reported that oligodendroglial-derived EVs are mainly uptaken by microglia and neurons but not by astrocytes and oligodendrocytes^7^. It is essential to identify the releasing cells (i.e., donor cells) and/or the target cells (i.e., recipient cells) of EVs circulating in the body not only to reveal the transfer mechanism of EVs among cells but also to use EVs as carriers for drug delivery.

We constructed a human CD63-copGFP gene regulated by the CAG promoter (CAG/human CD63-GFP). The CD63 protein is a member of the transmembrane 4 superfamily (tetraspanin), and is known as an EV marker. CD63 is highly enriched in late endosomes (MVBs) via an intracellular pathway from the trans-Golgi network or via endocytosis from the cell surface^8^. Within MVBs, CD63 is incorporated into intraluminal vesicles, through a trafficking pathway that requires ceramide in some types of cells^9^,^10^; then, the vesicles rich in CD63 are released as exosomes by fusion with the plasma membrane. In previous studies, a GFP-tagged CD63 gene (CD63-GFP) has been transfected into cultured cell lines to detect EV transfer and incorporation into recipient cells^11^,^12^. In addition, CD63-GFP *in vivo* transfection into the fruit fly (*Drosophila melanogaster*) has been reported; this study has shown that CD63-GFP-labelled EVs from the male reproductive glands mediate the interaction between sperm and the female reproductive tract after mating^13^. The present study is the first report of EVs labelled with CD63-GFP in mammals. We showed that exogenous human CD63-GFP expression was detected in the tissues of Tg rats by immunostaining and biochemical detection. Furthermore, the human CD63-GFP labels were detected in EVs isolated from the blood serum, breast milk, and amniotic fluid (AF) of the Tg rats. Our animal model may potentially provide a new approach to study the intraindividual transfer and/or mother-child transfer of EVs *in vivo*.

## Results

### Production of CD63-GFP rats and phenotypic analysis

**Figure 1:**
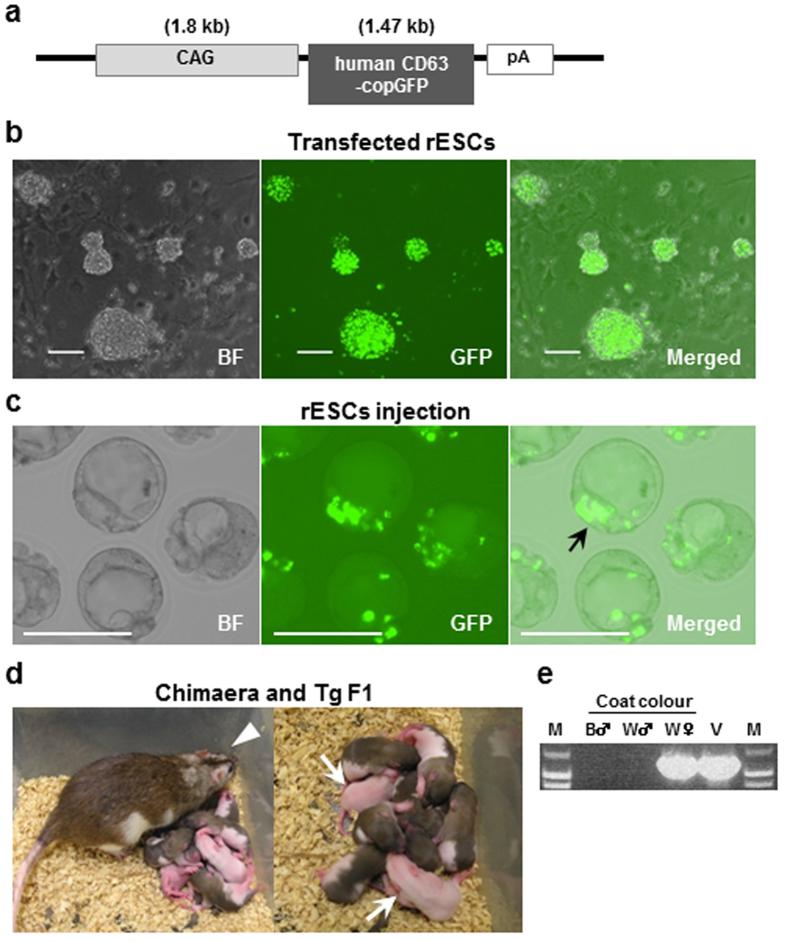
Generation of CAG/human CD63-GFP transgenic (Tg) rats. (**a**) Structure of the transgene construction. The transgene was constructed using human CD63-copGFP under control of the CAG promoter. (**b**) Image of rat embryonic stem cells (rESCs) transfected with the CAG/human CD63-GFP gene. The cultured rESCs expressed GFP. (**c**) Blastocysts after microinjection of the transfected rESCs. The arrow indicates rESC adherence to the inner cell mass (ICM). BF: bright field. Scale bars = 100 μm. (**d**) Adult female chimaeric rat from Wister-derived rESC (white-coated) injection into LEA blastocysts (brown-coated). White patches were present in the face (arrowhead). Two Tg offspring (white-coated) from mating a female chimaeric rat with a Wistar wild type (Wt) male (arrows). (**e**) Genotyping by PCR analysis of the extracted DNA from ear snips of the offspring. B: brown coat colour, W: white coat colour, V: CAG/human CD63-GFP vector, and M: size marker.

CAG/human CD63-GFP transgenic rats expressing copGFP-fusion protein to human CD63 under the control of the CAG promoter (Fig. 1a) were generated by gene transfection into rat embryonic stem cells (rESCs) (Fig. 1b) and injection of transfected rESCs into blastocysts (Fig. 1c). We selected the rESC colonies expressing GFP fluorescence, and further selected the rESC lines showing highly fluorescent signals that were comparatively stable after several passages. To generate coat-colour chimaeras, the transfected rESCs, which originated from a Wistar strain (white-coated), were injected into blastocysts from LEA x LEA mating (brown-coated) or LEA x Wistar mating (brown-coated). We observed that the rESCs were incorporated into the inner cell mass of the blastocysts (arrow in Fig. 1c). The white coat colour from the rESCs appeared mainly in the face (arrowhead in Fig. 1d). The chimaeric rats produced white-coated newborns after mating with Wistar males, showing the successful germline transmission of the rESCs (arrows in Fig. 1d). Furthermore, the transgenic offspring were genotyped using PCR analysis (Fig. 1e). The germline chimaera in this study was confirmed in one of four rESC lines, and it was named the Wistar-esTgN(CAG/CD63-GFP)3NCCRI strain. Tg male offspring were not viable after birth. Tg male foetuses, compared with Tg female foetuses, showed relatively high-level expression of human CD63-GFP in fluorescence microscopy observations (GFP++; Supplementary Fig. 1a) and western blotting analysis (anti-human CD63 and anti-copGFP; Supplementary Fig. 1b,c). There were no apparent developmental defects during gestation, and nontransgenic and transgenic male litters showed equal Mendelian ratios by genotyping: six males were GFP-negative (No. 2–7), and six males were GFP-positive (No. 1–6) in one litter (Supplementary Fig. 1b); three males were GFP-negative (No. 3–5), and three males were GFP-positive (No. 3, 5, 6) in another litter (Supplementary Fig. 1c). The Tg female rats were fertile, but they were likely to die sooner than usual, after reaching an age of four to six months. We also generated other Tg rats with a human CD63-GFP gene regulated by the *Sox2* promoter activated in neural stem cells because the CAG promoter is ubiquitously expressed. The tissue-specific human CD63-GFP Tg rats gave birth to both sexes; these rats were fertile and did not tend to die early (unpublished).

**Figure 2:**
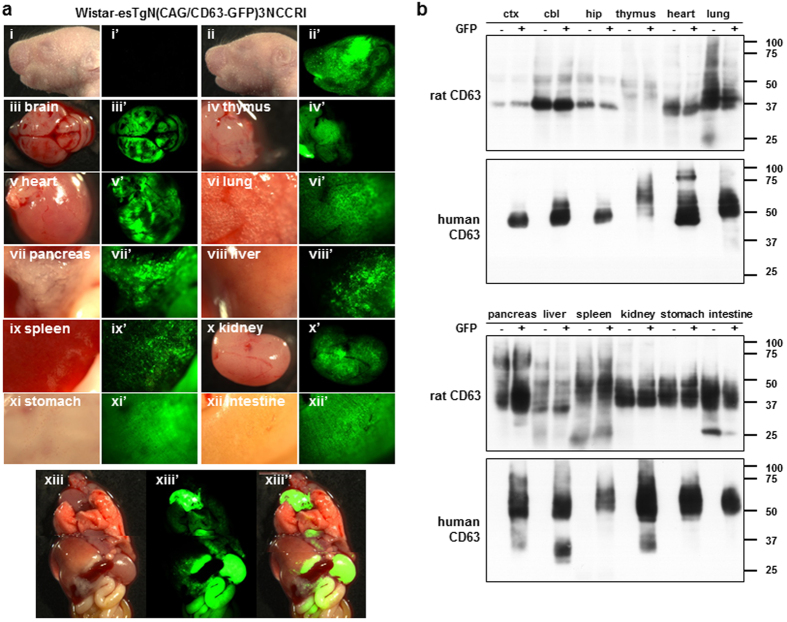
Human CD63-GFP expression analysis in Tg rats (Wister-esTgN(CAG/CD63-GFP)3NCCRI). (**a**) Pictures of main organs from Tg offspring (i–xiii: bright field, i’–xiii’: GFP, and xiii”: merged). GFP-negative (i and i’) and GFP-positive (ii and ii’) offspring were littermate. The heart, kidneys and stomach showed especially high fluorescent signals (xiii”). (**b**) Western blotting for endogenous rat CD63 and exogenous human CD63 in tissue lysates from GFP-negative (GFP−) and GFP-positive (GFP+) offspring. Ctx: cortex, cbl: cerebellum, and hip: hippocampus.

GFP-positive neonatal rats expressed exogenous human CD63-GFP throughout their bodies (Fig. 2a,b). The endogenous rat CD63 was ubiquitously expressed in main organs except for the thymus and liver, which showed expression signals that were lower and indistinct relative to those of other tissues (Fig. 2b). The heart, kidney and stomach expressed particularly high levels of human CD63-GFP (Fig. 2a xiii” and Fig. 2b). At embryonic day 18–19 (E18–19), GFP fluorescence was also observed in placentas (arrows in Supplementary Fig. 1a). Interestingly, the placentas of GFP-negative foetuses expressed GFP signals only on the maternal side (arrowhead in Supplementary Fig. 1a).

### CD63-GFP signals are located in endosomes, and extracellular secretion was attenuated by GW4869

**Figure 3:**
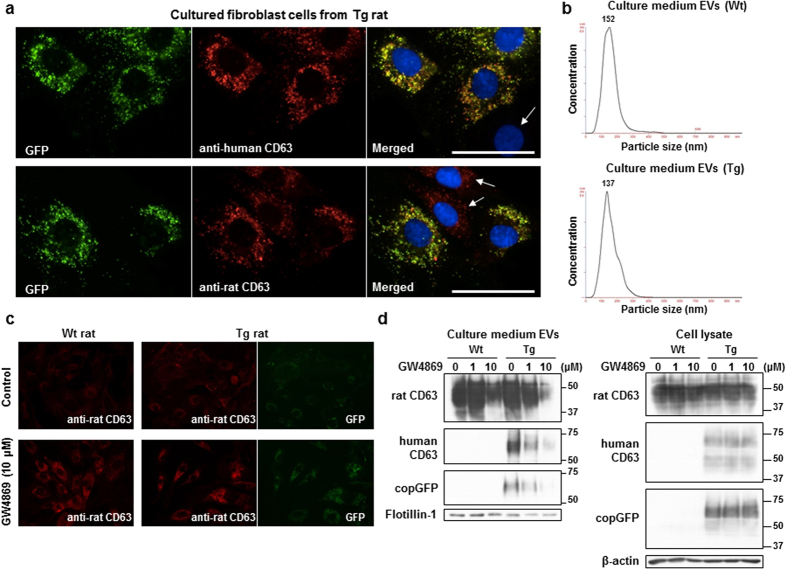
Human CD63-GFP localization and extracellular vesicle (EV) analysis in the primary fibroblast cells obtained from the caudal vertebrae of Tg rats. (**a**) Localization of human CD63-GFP in the cultured Tg rat cells. Immunostaining indicated the co-localization of GFP with human CD63 (upper panels) and with rat CD63-positive signals (lower panels) around nuclei (blue). Scale bars = 50 μm. (**b**) Size distribution of the EVs isolated from the conditioned medium of Wt and Tg rat cells was determined using a NanoSight system. (**c**,**d**) The relationship between ceramide and the secretion of EVs. The intracellular rat CD63-positive signals and GFP signals were increased after treatment with 10 μM GW4869, a neutral sphingomyelinase (nSMase) inhibitor, for 24 hours (**c**). Western blotting showed a GW4869-dependent decrease of EV markers (rat CD63 and flotillin-1) and human CD63-GFP in the isolated EVs (**d**; left). However, the expression levels of rat CD63 and human CD63-GFP in the cell lysates were not changed by GW4869 (**d**; right). β-actin was used as a loading control. Both generation and protein composition of the EVs did not show an apparent change by CD63-GFP overexpression (Supplementary Fig. 5).

To analyse the human CD63-GFP localization inside cells, primary fibroblast cells were prepared from caudal vertebrae of Wistar wild type (Wt) and Tg rats. Human CD63-GFP expression was located close to nuclei (upper panels in Fig. 3a), and GFP signals overlapped with endogenous rat CD63-positive signals (lower panels in Fig. 3a). The cells with low GFP expression showed no human CD63-positive signals, only rat CD63-positive signals (arrows in Fig. 3a). Time-lapse imaging of cells from Tg rats showed the movement of small GFP-positive organelles between plasma membranes and around nuclei (Supplementary Fig. 2). We isolated EVs from the conditioned medium (EV-free FBS) of the cultured fibroblast cells by ultracentrifugation. Both EVs from Wt and Tg cells showed a peak in particle size between 100 and 200 nm (Wt; 152 nm and Tg; 137 nm) by measurement using a NanoSight system (Fig. 3b). It has been suggested that there are multiple molecular machineries involved in the generation of MVB and the sorting in endosomes have different manners (e.g. ceramide-dependent or -independent), and which mechanism predominates depends on the cell type^1^,^14^. The tetraspanin CD63 plays in the MVB formation and the endosomal sorting^14^,^15^. Next, to examine whether the extracellular secretion of human CD63-GFP proteins was regulated via the same biogenesis pathway as endogenous rat CD63-containing exosomes, the fibroblast cells were treated with a neutral sphingomyelinase (nSMase) inhibitor, GW4869, which is known to block ceramide biosynthesis and reduce EV secretion^16^. The rat CD63 signals inside the Wt and Tg cells increased after incubation with 10 μM GW4869 for 24 hours, and the GFP fluorescence signals were also increased in Tg cells after GW4869 treatment (Fig. 3c). The expression levels of rat CD63 and human CD63-GFP proteins in the cell lysates remained unchanged after GW4869 treatment (right in Fig. 3d). In contrast, the extracellular rat CD63 and human CD63-GFP levels in the EVs collected by ultracentrifugation decreased with GW4869 in a dose-dependent manner (left in Fig. 3d). The levels of another EV marker, flotillin-1, were also reduced in Wt (10 μM GW4869) and Tg cells (1 and 10 μM GW4869) compared with cells that did not receive GW4869. These results suggested that GW4869 blocked EV release into the conditioned medium but did not affect protein expression. These results also suggested that the exogenous human CD63-GFP in fibroblast cells was secreted through the ceramide-dependent biogenesis pathway, as was endogenous rat CD63.

### The EVs labelled with CD63-GFP from rat body fluids

**Figure 4:**
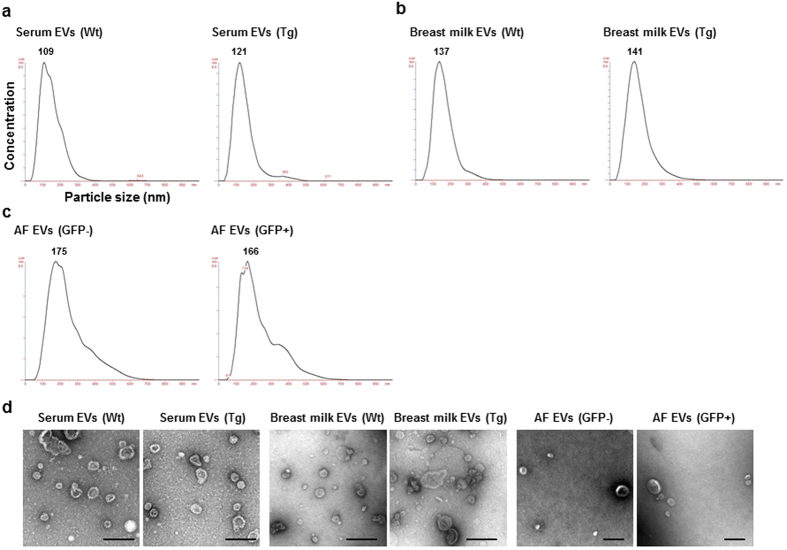
Characterization of the EVs isolated from three body fluids of Wt and Tg rats. (**a**–**c**) Size distribution of EVs from serum (**a**), breast milk (**b**) and amniotic fluid (AF) (**c**) determined using a NanoSight system. (**d**) Electron microscopy (EM) images of EVs. Scale bars = 200 nm. AF samples were collected from pregnant Tg rats at embryonic day 16–17 (E16–17) after mating with Wt males. GFP−: GFP-negative foetuses. GFP+: GFP-positive foetuses.

**Figure 5:**
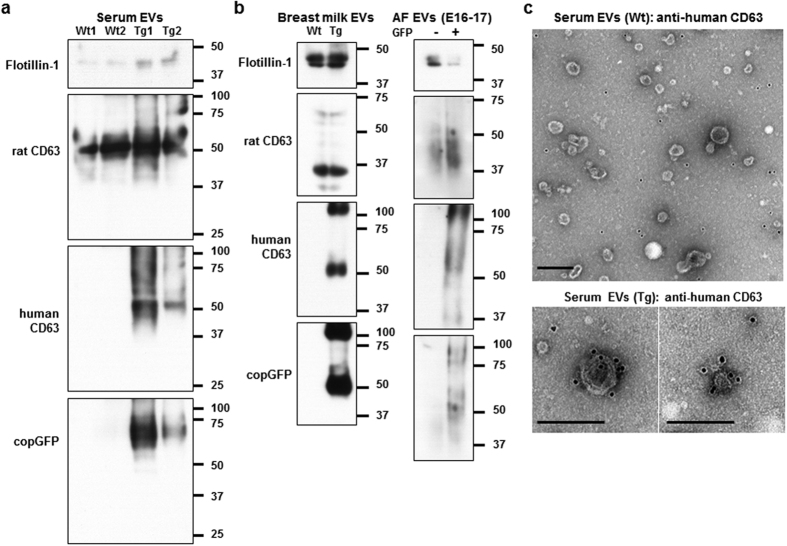
Analysis of the EVs labelled with human CD63-GFP in the three body fluids. (**a**,**b**) Western blotting analysis of the EVs isolated from serum (**a**), breast milk and AF (**b**) of Wt and Tg rats for flotillin-1, rat CD63, human CD63 and copGFP. AF samples were collected from pregnant Tg rats at E16–17 after mating with Wt males. GFP−: GFP-negative foetuses. GFP+: GFP-positive foetuses. (**c**) Immunoelectron microscopy images of serum-derived EVs from Wt and Tg rats using anti-human CD63 antibody (10 nm gold particles). Scale bars = 200 nm.

EVs were isolated from blood serum, breast milk and AF of Wt and Tg rats using ultracentrifugation methods. Nanoparticle tracking analysis showed a peak between 100 and 200 nm in each sample (Fig. 4a–c). The particles imaged by electron microscopy (EM) were observed to be mainly between 50–150 nm in size (Fig. 4d). Western blot analysis revealed that the EVs of the three body fluids from Wt and Tg rats were positive for EV markers, flotillin-1 and endogenous rat CD63 (Fig. 5a,b). Furthermore, signals of antibodies to human CD63 and copGFP in the EVs were detected in the three body fluids of Tg rats but not in those of Wt rats (Fig. 5a,b). To visually confirm the presence of EVs labelled with human CD63-GFP, immunoelectron microscopy analysis was performed using serum-derived EVs. The EVs from Wt rats were not labelled with anti-human CD63 antibody (Fig. 5c, upper and Supplementary Fig. 3a). In contrast, some of the EVs from Tg rats were immunolabelled with anti-human CD63 antibody, and the immunolabelling for human CD63 was detected on EVs 50–100 nm in sizes (Fig. 5c, lower and Supplementary Fig. 3b–d). The negative staining without the first antibody showed no immunoreactivity on the Tg EVs (Supplementary Fig. 3e). These results suggested that human CD63 was enriched in the subpopulation of EVs in Tg rats.

### Transfer of the labelled EVs with CD63-GFP into recipient cells *in vitro*

**Figure 6:**
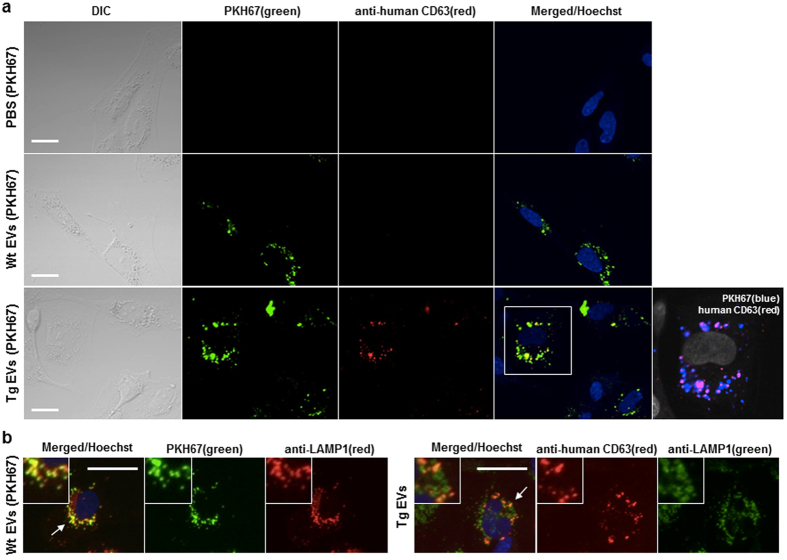
Uptake of serum-derived EVs by rat embryonic fibroblast cells (REFs). (**a**) EVs of Wt rats, Tg rats and vehicle PBS (−) as a control were prelabelled with PKH67. Then, REFs were incubated with PKH67-labelled EVs for 11 hours. The incorporated EVs in the recipient REFs were detected by PKH67 (green) and anti-human CD63 (red). The Tg EVs are also shown as magnified views (white square region) with different colours (PKH67: blue, human CD63: red, nuclei: gray). Scale bars = 20 μm. (**b**) Images of co-localization of EVs with LAMP1 in REFs. EVs of Wt (with PKH67) and Tg rats were incubated with REFs for 10 hours, and the EVs from Tg rats were detected using an antibody to human CD63. Magnified views (arrows) are shown in the insets. Scale bars = 25 μm. Nuclei were counterstained with Hoechst 33342 (blue).

To evaluate the utility of the labelled EVs from the body fluids of Tg rats for monitoring EV transfer between cells, the serum-derived EVs were incubated with primary rat embryonic fibroblast cells (REFs) *in vitro* for 10–11 hours (Fig. 6a,b). Although it was difficult to chase the incorporated Tg EVs by GFP fluorescent directly, the Tg EVs in the recipient cells were detectable by immunostaining with antibodies to human CD63 and copGFP (Fig. 6a and Supplementary Fig. 4). In general, the EVs collected by ultracentrifugation consists of microvesicles and exosome subpopulations, and the immunoelectron microscopy analysis in this study indicated human CD63-immunopositive and –immunonegative subpopulations of the EVs in the serum of Tg rats. To examine how the EVs with human CD63-GFP were transferred into REFs, Wt and Tg EVs were prelabelled with PKH67, a green fluorescent lipid dye that stains all EV subpopulations. We did not observe green fluorescent signals in the cells that were incubated with vehicle PBS (−) labelled with PKH67. In contrast, PKH67-labelled EVs showed highly intense fluorescent signals around cells nuclei (Fig. 6a). The human CD63 immunosignals were detected only in Tg EVs and a substantial proportion of EVs incorporated into the recipient cells were human CD63-immunnopositive (Fig. 6a). Furthermore, late endosome/lysosome marker LAMP1 was colocalized to both PKH67 and human CD63 in the recipient REFs (arrows in Fig. 6b). These results demonstrate a substantial uptake of CD63-enriched EVs by the recipient cells through the endocytic pathway.

## Discussion

EVs are released into the extracellular environment, and they have been found in various body fluids, such as CSF^17^, breast milk^18^, saliva^18^, urine^19^, AF^20^, and blood serum. The results of proteome and miR profiling have shown that the EVs from body fluids carry many immune- or development-related components. Furthermore, the analysis of EVs from the blood of mothers during pregnancy^21^ and from breast milk during lactation^22^ have provided data showing time-dependent changes in the number of EVs or RNA contents of EVs. However, the studies of EV transfer using EVs isolated from body fluids have been very few in number compared with the experiments using EVs isolated from *in vitro* culture. Most of these studies injected labelled EVs that were collected from conditioned medium of cultured cells into a body to examine the transfer of EV contents^23^. PKH67 and PKH26 are the most common reagents used to stain EVs. Because they are membrane dyes with a high affinity for lipids, all EVs are labelled with PKH reagents. In addition, body fluids include many more lipid components than conditioned medium, and these lipids may also be stained by PKH reagents. In this study, we generated and analysed a novel Tg rat expressing human CD63-GFP under the CAG promoter. Human CD63-GFP expression was detected by western blotting analysis in the EVs collected from three body fluids: blood serum, breast milk and AF. CD63 proteins are highly enriched particularly on exosome membranes via trafficking into late endosomes (MVBs)^8^. We expected that, in combination with other EV markers, human CD63-GFP might be helpful to distinguish EVs from other vesicles and lipid components as well as provide more precise identification of the EVs endocytosed into recipient cells.

This study suggested that the overexpression of CD63 had strong functional effects *in vivo*, which resulted in embryonic lethality for males and premature death for females. It has been reported that CD63 associates with many protein, and has a key role in intracellular trafficking and cell signalling activity, which indicates that CD63 binding to other membrane proteins regulates various cellular processes, such as cell migration, adhesion and differentiation, via altering complex formation at the plasma membrane^8^,^24^,^25^,^26^. In mast cells (MCs), the absence of CD63 results in significantly decreased MC degranulation, which led to a reduction of allergic reactions^27^. The Tg rats in this study often exhibited inflammation around the eyes as animals grew (data not shown). Therefore, we considered the possibility that the Tg rats might overexpress CD63 in MCs, which in turn might causing allergic inflammation by increased MC degranulation. We generated another line of Tg rats expressing human CD63-GFP under the *Sox2* promoter, which expressed it in the kidney in addition to the brain; a large amount of urine output was observed, but no lethality or infertility was observed in either gender (unpublished). A previous report has found that, although CD63-deficient mice display no lethality or infertility due to compensation by other tetraspanins, they exhibit morphologic changes in the kidney, and increased urine output^28^, similarly to that of our Tg rats. These results suggest that CD63-GFP will be further useful for visualizing EVs using tissue- or stage-specific expression constructs, with consideration of the potential effects on phenotype.

While the present experiments did not show that serum-derived EVs from the Tg rats retained GFP fluorescence after transfer into recipient cells via *in vitro* assay, the EV signals in recipient cells were clearly detectable by using an antibody to human CD63. The immunoelectron microscopy analysis revealed that the immunolabelling with human CD63-specific antibody was not detected on all isolated EVs. In addition, the transfer of EVs into recipient cells *in vitro* showed the human CD63 immunoreactivity in many EVs. These results suggested that human CD63-GFP labelling would provide a helpful tool for tracing of incorporated exosomes although more detailed experiments are required. This animal model is intended to contribute to research on the transition pathway of EVs using body fluids. Several studies on breast milk have suggested that the composition of milk-derived EV may be a potential immune-regulator for infants^29^,^30^ and an activator of postnatal growth^31^. Immune-related miRs have been identified in the milk-derived EVs^29^,^32^. Other than miRs, the milk-derived EVs carry bioactive TGF-β^33^, which is known to differentiate naïve T cells into regulatory T cells^34^ and to promote development of the intestinal barrier^35^. Nevertheless, the transfer pathway and recipient cells of milk-derived EVs in infants remain poorly understood. A previous report has found that mouse intestinal stem cells show uptake of food (grape)-derived EV-like vesicles that were stained by PKH dye *in vivo*, and the targeted cells exhibit increased proliferation after taking up the grape-derived vesicles^36^. Our Tg rats produced the EVs labelled with human CD63-GFP, which were detected only in the tissues of Tg rats and not in those of Wt rats. We expect that study of *in vivo* uptake of the milk-derived EVs from Tg rats will reveal interesting targets in the intestinal tissues of infants.

The novel rat model that we generated allowed studies of the movement of endosomes before EV release and the detection of EVs released. Our new animal model shows promise for facilitating further studies to reveal the communication between cells/tissues via EVs.

## Methods

### Animals

All animals (Wistar, LEA and Tg (Wistar-esTgN(CAG/CD63-GFP)3NCCRI) rat strains) used in this study were treated in accordance with the institutional guidelines of the Animal Ethics Committee for the care and use of animals in the National Institute of Neuroscience, National Centre of Neurology and Psychiatry, and National Cancer Centre Research Institute, Japan.

### Production of CAG/human CD63-GFP Tg rats using rat ES cells

A DNA fragment encoding human CD63-copGFP (human CD63-GFP) from a pCT-CMV-CD63-GFP vector (System Biosciences, CA, USA) was subcloned into a pECFP-1 plasmid (Clontech, Shiga, Japan) containing CAG promoter. The plasmid CAG/human CD63-GFP was linearized by SalI and was then transfected into Wistar rESCs using a Mouse ES Cell Nucleofector Kit (Lonza, Basel, Swiss) and an Amaxa Nucleofector (A-013 program) as described previously^33^. pCAG/human CD63-GFP contained a neomycin/kanamycin selection cassette. The rESCs were seeded onto mitomycin-C-treated neomycin-resistant MEFs (Millipore, MA, USA) in YPAC medium^33^ with 2% Matrigel (Becton Dickinson, NJ, USA). Then, G418 (Geneticin) (Sigma-Aldrich, MO, USA) was added to the culture medium at day 1 for the selection of transfected cells. Colonies showing GFP fluorescence were selected and expanded. The rESCs in this study were derived from the cell lines established by Kawamata and Ochiya^37^.

Blastocysts were collected at 4.5 days post coitus (dpc) from LEA x LEA or Wistar x LEA mating and injected with approximately 12 transfected rESCs. The injected blastocysts were surgically transferred into the uterine horns of 3.5 dpc pseudopregnant Wistar rats. Chimaeric rats were identified by coat colour. Germline transmission was confirmed by the coat colour of F1 rats resulting from mating with Wistar rats. Transgenic offspring were genotyped by GFP fluorescence and PCR analysis. Sex determination in foetuses was performed by PCR using the male-specific *Sry* gene as a marker.

### Immunoblotting

For total protein extraction, the tissues and cultured cells were solubilized in lysis buffer (1% SDS, 10 mM Tris-HCl pH 7.5, 5 mM EDTA, 10 mM sodium pyrophospate, 10 mM NaF, 1 mM PMSF and 2 mM Na_3_VO_4_). The samples were sonicated on ice, and then the lysates were cleared by centrifugation. Protein concentration was measured using a Pierce BCA Protein Assay Kit (Life Technology, CA, USA).

Cell lysates and EVs were separated by SDS-polyacrylamide gel electrophoresis (SDS-PAGE) and were transferred onto Immobilon-P Transfer membranes (Millipore). After being blocked with 5% skim milk, the membranes were probed with primary antibodies specific to the following proteins: rat CD63 (1:250; AbD Serotec, CA, USA); human CD63 (1:250; Becton Dickinson); copGFP (1:10000; Evrogen, Moscow, Russia); and flotillin-1 (1:500; Becton Dickinson). β-actin (1:5000; Sigma-Aldrich) was used as a loading control. The membranes were subsequently incubated with secondary antibodies, and the signals were detected using chemiluminescent reagents (ImmunoStar and ImmunoStar LD, Wako, Tokyo, Japan).

### Immunocytochemistry

Cultured cells were fixed with 4% paraformaldehyde (PFA) for 15–20 min at room temperature. After being washed, cells were incubated with primary antibodies in blocking solution containing 0.2% Triton X-100 (Sigma-Aldrich) and 10% FBS in PBS at 4 °C overnight. Primary antibodies specific to the following proteins were used: rat CD63 (1:200; AbD Serotec) and human CD63 (1:200; Becton Dickinson). After the cells were stained with Alexa Fluor 546 Goat anti-Mouse IgG_1_ antibody (1:2000; Life Technologies), they were imaged with BIOREVO BZ-9000 (Keyence, Osaka, Japan) and Axiovert 200 (Carl Zeiss, Westlar, Germany) fluorescence microscopes. DAPI was used to stain cell nuclei.

### Fibroblast culture and inhibition of EV secretion

Primary fibroblast cells were prepared from caudal vertebrae of Wt and Tg rats (CAG/human CD63-GFP) at postnatal day 21 (P21). The primary cells were maintained in DMEM (Life Technologies) with 10% FBS and antibiotic-antimycotic solution (Life Technologies) at 37 °C in 5% CO_2_.

For inhibition of EV secretion, the fibroblast cells were plated on glass-bottom dishes and 10 cm culture dishes; after expansion, GW4869 (Sigma-Aldrich) treatments were performed for 24 hours. The 35 mm glass-bottom dishes were used to observe intracellular rat CD63 as an EV marker and human CD63-GFP expression, and cells on the 10 cm dishes were stored at −80 °C until protein extraction. Conditioned medium from the 10 cm dishes was used for the isolation of EVs. To isolate EVs, conditioned medium from the 10 cm dishes was replaced with the EV-free FBS (System Biosciences) medium before GW4869 treatment. Culture supernatant was centrifuged at 1,400 rpm for 10 min and then filtered through a 0.22 μm membrane filter (Millipore) to remove cellular debris. EVs were collected by ultracentrifugation at 35,000 rpm for 70 min at 4 °C using a Beckman SW41Ti rotor (Beckman, CA, USA). The pellets were washed with PBS by re-ultracentrifugation and were resuspended in PBS. The isolated EVs were quantified by size and count using a NanoSight system (NanoSight, Amesbury, UK) and by measuring their protein concentration using a Pierce BCA Protein Assay Kit.

### EV isolation from serum, breast milk and AF body fluids

Blood samples were collected from Wt and Tg adult female rats. The blood serum samples were centrifuged at 1,400 rpm for 10 min and then at twice at 10,000 g for 15 min to remove blood cells; this was followed by ultracentrifugation at 35,000 rpm for 70 min at 4 °C. Breast milk samples were collected from the stomachs of GFP-negative newborns (P4-5) taking milk from Wt or Tg mother rats. Collected solid milk samples were homogenized and resuspended in PBS; then, they were centrifuged at 2,000 g for 10 min to remove fat and cell debris. Non-transparent supernatants were filtered through a 0.22 μm filter to remove residual fat, producing a clear supernatant (milk whey). Finally, the EVs were collected by ultracentrifugation. AF samples were collected from pregnant Tg rats at E16-17 that had mated with Wt males. Tg foetuses were identified by the fluorescence of GFP under a fluorescence stereomicroscope (Leica, Wetzlar, Germany). AF samples were centrifuged at 2,000 g for 10 min and then at 10,000 g for 10 min, and EVs were collected by ultracentrifugation.

### EM analyses

The body fluid-derived EVs were plated on collodion-carbon-coated grids and fixed with 2% PFA. For immunoelectron microscopy, the serum-derived EVs were permeabilized with 0.1% saponin, stained with anti-human CD63 (1:50, Becton Dickinson), and then incubated in secondary anti-mouse antibody conjugated with 10-nm gold particles (Amersham, Buckinghamshire, UK). Finally, the EVs were post-fixed in 0.2% glutaraldehyde and negatively stained with uranyl acetate. The images were captured using a transmission electron microscope (Tecnai Spirit; FEI, OR, USA).

### Transfer of serum EVs into REFs

REFs were prepared from Wt rats at E14.5. Embryos were dissociated by trypsin treatment (Sigma-Aldrich), and the single cells were cultured in DMEM including 10% FBS and antibiotic-antimycotic solution at 37 °C in 5% CO_2_. Serum-derived EVs from Wt or Tg rats were isolated using ultracentrifugation. The EVs were visualized using a PKH67 fluorescence labelling kit (Sigma-Aldrich). Serum EVs were incubated with REFs on 24-well glass bottom plates for 10–11 hours at 37 °C in 5% CO_2_. After being washed, the cultured cells were fixed with 4% PFA. The EVs from Tg rats were detected using anti-human CD63 antibody after having been transferred into REFs. To analyse localization of EVs in REFs, anti-LAMP1 antibody (1:250, Sigma-Aldrich) was used. After the cells were stained with Alexa Fluor 546 Goat anti-Mouse IgG_1_ (1:2000; Life Technologies), Alexa Fluor 546 Goat anti-Rabbit IgG (1:2000; Life Technologies) and Alexa Fluor 488 Goat anti-Rabbit IgG (1:200; Life Technologies) antybodies, internalization of EVs were captured with a FV1000 confocal microscope (OLYMPUS, Tokyo, Japan) and a BIOREVO BZ-9000 fluorescence microscopy (Keyence, Osaka, Japan). Hoechst 33342 was used to stain cell nuclei.

All experimental protocols were approved by the National Institute of Neuroscience, National Center of Neurology and Psychiatry, and National Cancer Centre Research Institute, Japan.

## Additional Information

**How to cite this article**: Yoshimura, A. *et al.* Generation of a novel transgenic rat model for tracing extracellular vesicles in body fluids. *Sci. Rep.* **6**, 31172; doi: 10.1038/srep31172 (2016).

## Supplementary Material

Supplementary Information

Supplementary Information
