# Supplementary material for: Generation of a novel transgenic rat model for tracing extracellular vesicles in body fluids
Source: Sci Rep. 2016 Aug 19;6:31172. doi: 10.1038/srep31172 (PMC4990884; doi:10.1038/srep31172)
Supplement: Supplementary Information [file srep31172-s1.docx]

**Title:**

Generation of a novel transgenic rat model for tracing extracellular vesicles in body fluids

**Authors:**

Aya Yoshimura, Masaki Kawamata, Yusuke Yoshioka, Takeshi Katsuda, Hisae Kikuchi, Yoshitaka Nagai, Naoki Adachi, Tadahiro Numakawa, Hiroshi Kunugi, Takahiro Ochiya & Yoshitaka Tamai

**Figure legends**

**Supplementary Figure 1**

**Expression analysis of human CD63-GFP in rat foetuses.** (**a**) Littermates at embryonic day 18-19 (E18-19) from mating a Tg female with a Wt male. The foetuses showed three different fluorescence intensities: GFP-negative (i, GFP-), GFP-highest positive (ii, GFP++) and GFP-lowest positive (iii, GFP+). Placenta (arrows) also showed GFP expression, but the fluorescent signals in GFP-negative foetuses were observed only on the maternal side (arrowhead). BF: bright field. Scale bars = 5 mm. (**b**,**c**) Genotyping and western blotting analysis using the samples extracted from the forelimbs of littermates at E18-19 (**b**) and E17 (**c**). The male-specific *Sry* gene was used as a marker for sex determination of foetuses. Proteins for rat CD63, human CD63 and copGFP were examined. β-actin was used as a loading control. M: size marker.

**Supplementary Figure 2**

**Time-lapse images of internalized CD63-GFP-positive organelles.** GFP signals in the primary fibroblast cells obtained from the caudal vertebrae of Tg rats were captured with an Axio Observer Z1 fluorescence microscope system (Carl Zeiss) (arrows). During time-lapse imaging, the medium was replaced with buffer (120 mM NaCl, 4 mM KCl, 1.2 mM KH_2_PO_4_, 2 mM CaCl_2_, 1 mM MgSO_4_, 30 mM glucose, and 20 mM N-(2-hydroxyethyl)piperazine-N’-2-ethanesulfonic acid; pH 7.4), which was incubated at 37 °C. Nuclei were stained with DAPI (blue). BF: bright field. Scale bars = 10 μm.

**Supplementary Figure 3**

**Immunoelectron microscopy images of serum-derived EVs.** (**a-d**) EVs obtained from Wt (a) and Tg (b-d) rats immunostained with anti-human CD63 antibody followed by gold particles. Arrows indicate the immunolabelled EVs. The EVs from Tg rats shown in Figure 5c are indicated by white squares. The first antibodies were used at 1/200 dilution (a,b) or 1/50 dilution (c,d). (**e**) The negative staining without anti-human CD63 antibody in the Tg rats. Scale bars = 200 nm.

**Supplementary Figure 4**

**Detection of Tg serum-derived EVs taken by REFs.** After REFs were incubated with the serum-derived EVs of Tg rats for 11 hours, the incorporated EVs were detected by antibodies for copGFP (1:500; Evrogen, top panels) and human CD63 (1:200; Becton Dickinson, middle panels). Double staining with copGFP and human CD63 showed co-localization of them (bottom panels). Scale bars = 20 μm.

**Supplementary Figure 5**

**Analysis of the generation and composition of EVs by overexpression of CD63.** (**a,b**) To assess the effects of CD63-GFP overexpression on the generation and composition of EVs in primary culture of Wt and Tg (CAG/human CD63-GFP) rats, we selected liver and rat embryonic fibroblasts (REFs) that express low and high levels of endogenous CD63, respectively. Western blotting analysis of the cell lysates (**a**) and the EVs collected from the cultured medium of hepatocytes (Hep) and REFs (**b**). The membranes were probed with primary antibodies specific to human CD63 (1:250; Becton Dickinson), copGFP (1:10000; Evrogen), rat CD63 (1:250; AbD Serotec), flotillin-1 (1:500; Becton Dickinson), Alix (1:500; Millipore), TSG101 (1:500; Santa Cruz Biotechnology, TX, USA) and LAMP1 (1:500; Sigma-Aldrich). The expression levels of five EV markers (endogenous rat CD63, flotillin-1, Alix, TSG101 and LAMP1) showed no significant difference between Wt and Tg cells (**a**). Furthermore, the protein composition in the EVs did not show an apparent change by CD63-GFP overexpression (**b**). N.D. = not detected.

Hep and REFs were from an adult female and foetuses at E14, respectively. Hep were isolated from 10‒11-week-old female rats using the procedure of Seglen (Seglen, 1976). Briefly, after preperfusion with Ca^2+^-free Hank’s/EGTA solution through the portal vein, the liver was perfused with approximately 400 mL of Hank’s solution containing 0.05% collagenase at 25-30 mL / min. The extracted liver was mechanically digested with a surgical knife and further digested in 0.025% collagenase solution at 37°C for 15 min. The digested liver was then filtered twice with sterilized cotton mesh, and the cell suspension was collected by centrifugation at 57 x g for 1 min. Large cell aggregates were eliminated by filtering the cell suspension with a 60 μm stainless double mesh cell strainer (Ikemoto Scientific Technology Co., Ltd., Tokyo, Japan), and the flowthrough was collected by centrifugation at 57 x g for 1 min. After removing dead cells using Percoll (GE Healthcare, WI, USA) by centrifugation at 57 x g for 10 min, the cells were washed in E-MEM twice by centrifugation at 57 x g for 2 min. The purified Hep were resupended in serum-free hepatocyte culture medium (HCM^TM^ BulletKit; Lonza, Walkersville, MD, USA) and seeded to collagen type I-coated 10 cm dishes (Asahi Technoglass, Shizuoka, Japan) at 1 x 10^5^ cells / cm^2^. The medium was replaced with fresh HCM on day 1. Following further 24 hours incubation (day 2), culture supernatant was harvested for EV preparation. Conditioned medium of REFs culture was replaced with the EV-free FBS medium 24 hours before collection of culture supernatant. Culture supernatant of Hep and REFs was centrifuged and then filtered through a 0.22 μm filter to remove cellular debris. EVs were collected by ultracentrifugation at 35,000 rpm for 70 min at 4°C. The generation of EVs was evaluated by counting particles using a NanoSight system and by protein concentration of the isolated EVs. There is no significant change in the number of EVs between Wt and Tg samples in both types of cells.

Reference

Seglen, P. O. Preparation of isolated rat liver cells. *Methods Cell Biol.* **13,** 29-83 (1976).
