# Supplementary material for: Generation of a novel transgenic rat model for tracing extracellular vesicles in body fluids
Source: Sci Rep. 2016 Aug 19;6:31172. doi: 10.1038/srep31172 (PMC4990884; doi:10.1038/srep31172)
Supplement: Supplementary Information [file srep31172-s2.pptx]

## Slide 1
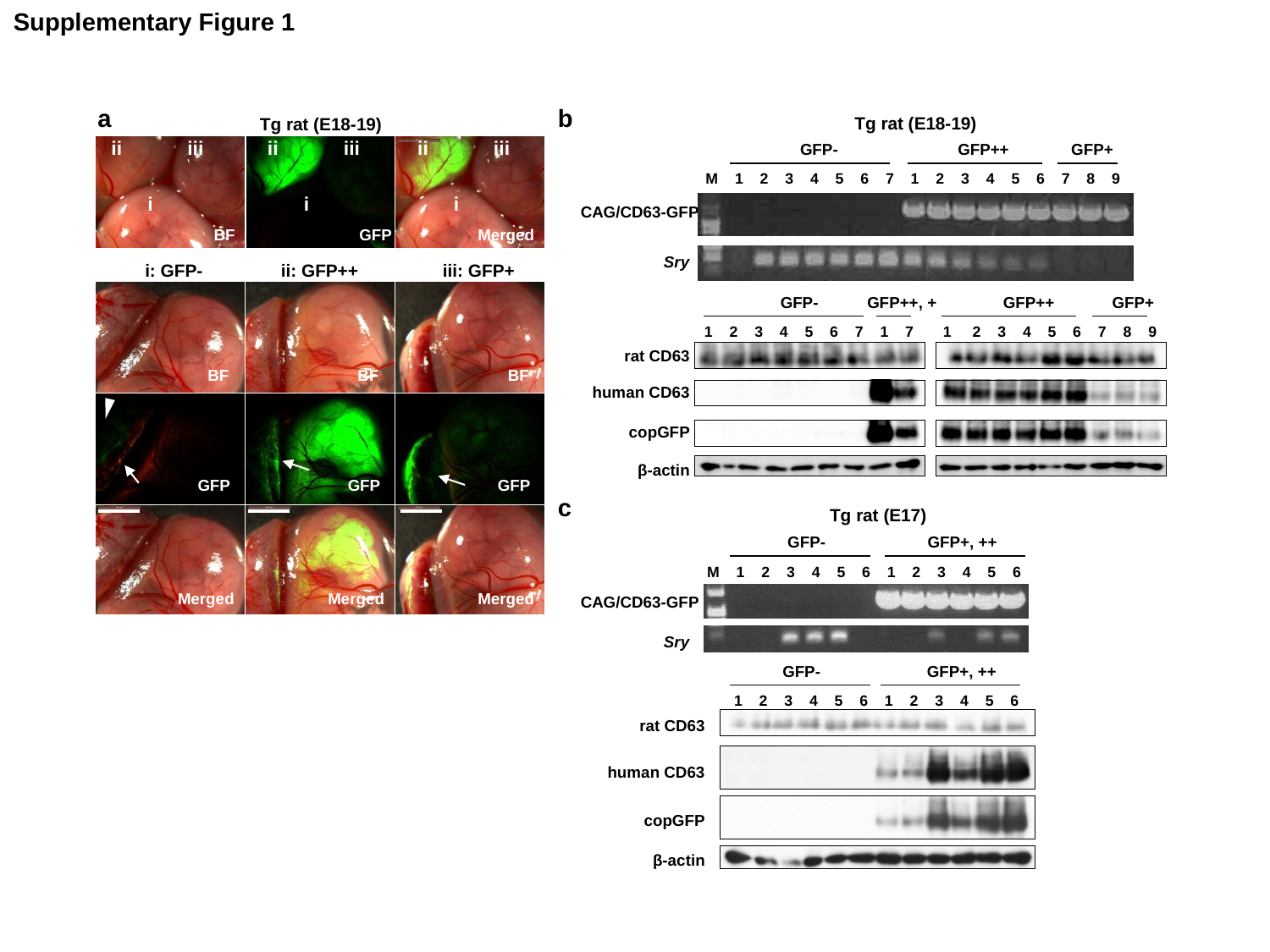

Supplementary Figure 1
a
b
Tg rat (E18-19)
Tg rat (E18-19)
ii
iii
i
ii
iii
i
ii
iii
i
GFP- GFP++ GFP+
M 1 2 3 4 5 6 7 1 2 3 4 5 6 7 8 9
CAG/CD63-GFP
BF
GFP
Merged
Sry
i: GFP-
ii: GFP++
iii: GFP+
GFP- GFP++, + GFP++ GFP+
1 2 3 4 5 6 7 1 7 1 2 3 4 5 6 7 8 9
rat CD63
BF
GFP
Merged
BF
GFP
Merged
BF
GFP
Merged
human CD63
copGFP
β-actin
c
Tg rat (E17)
GFP- GFP+, ++
M 1 2 3 4 5 6 1 2 3 4 5 6
CAG/CD63-GFP
Sry
GFP- GFP+, ++
1 2 3 4 5 6 1 2 3 4 5 6
rat CD63
human CD63
copGFP
β-actin

## Slide 2
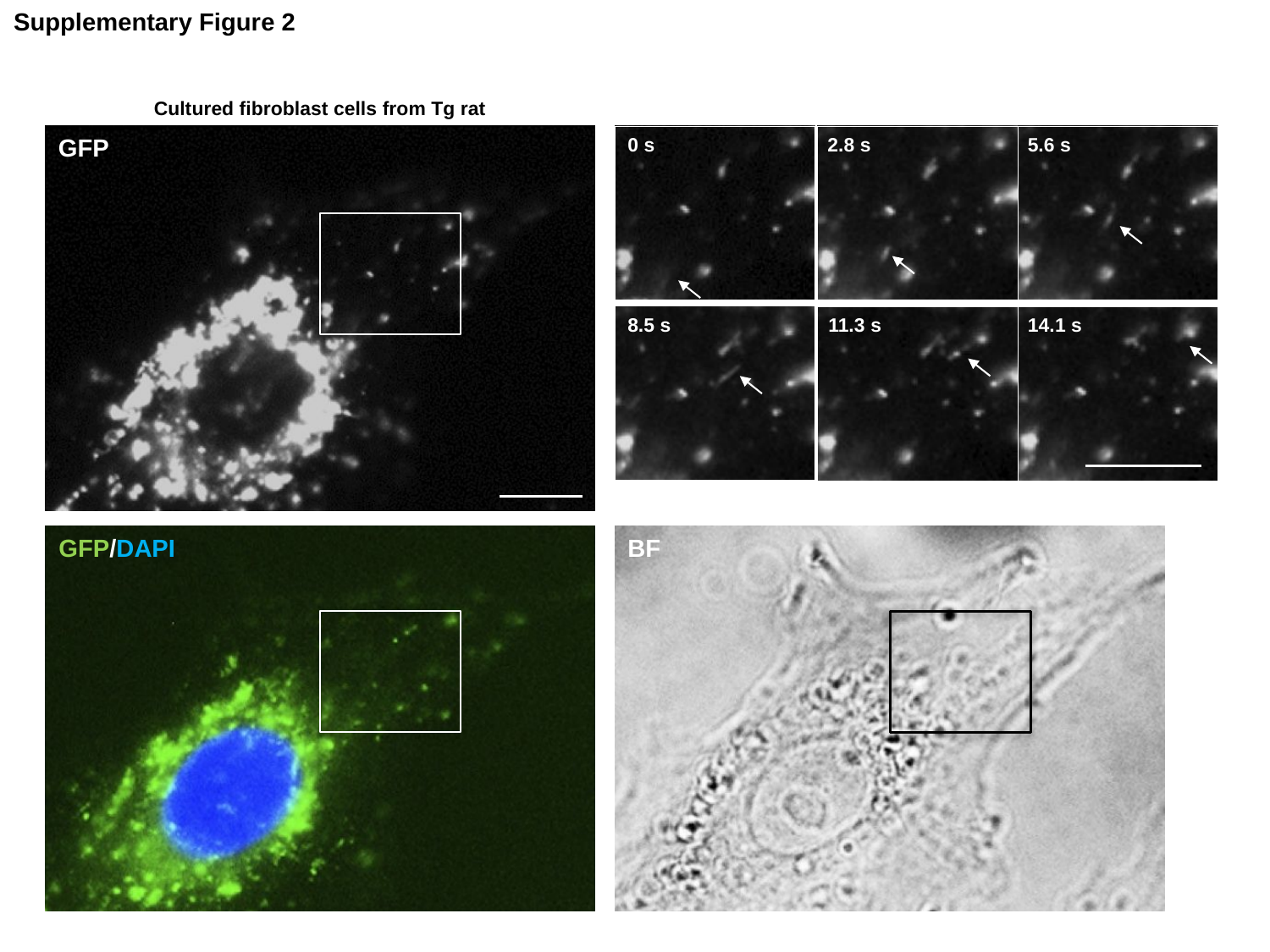

Supplementary Figure 2
Cultured fibroblast cells from Tg rat
GFP
0 s
2.8 s
5.6 s
8.5 s
11.3 s
14.1 s
GFP/DAPI
BF

## Slide 3
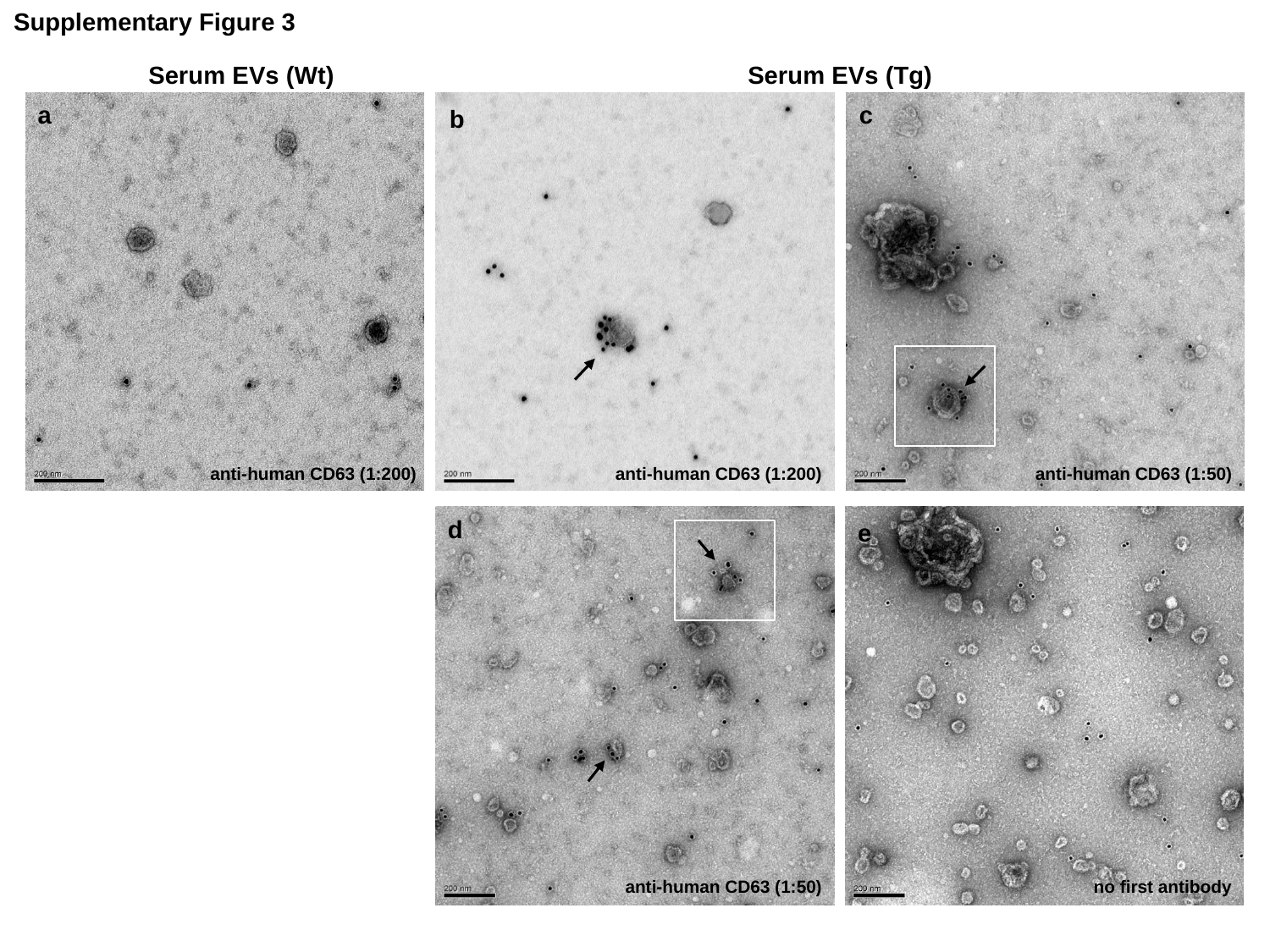

Supplementary Figure 3
Serum EVs (Wt)
Serum EVs (Tg)
a
b
c
anti-human CD63 (1:200)
anti-human CD63 (1:200)
anti-human CD63 (1:50)
d
e
anti-human CD63 (1:50)
no first antibody

## Slide 4
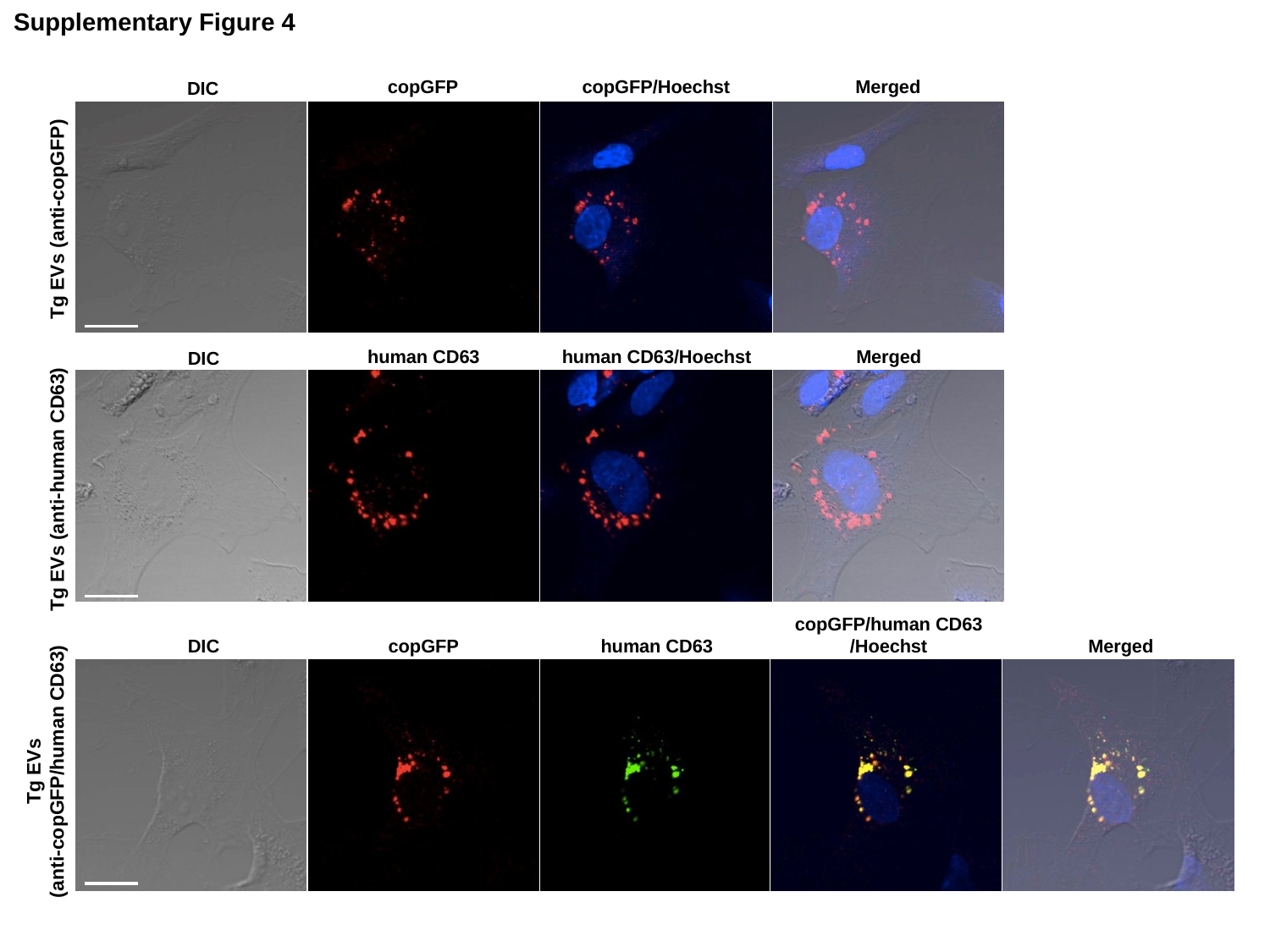

Supplementary Figure 4
copGFP
copGFP/Hoechst
Merged
DIC
Tg EVs (anti-copGFP)
human CD63
human CD63/Hoechst
Merged
DIC
Tg EVs (anti-human CD63)
copGFP/human CD63
/Hoechst
DIC
copGFP
human CD63
Merged
Tg EVs
(anti-copGFP/human CD63)

## Slide 5
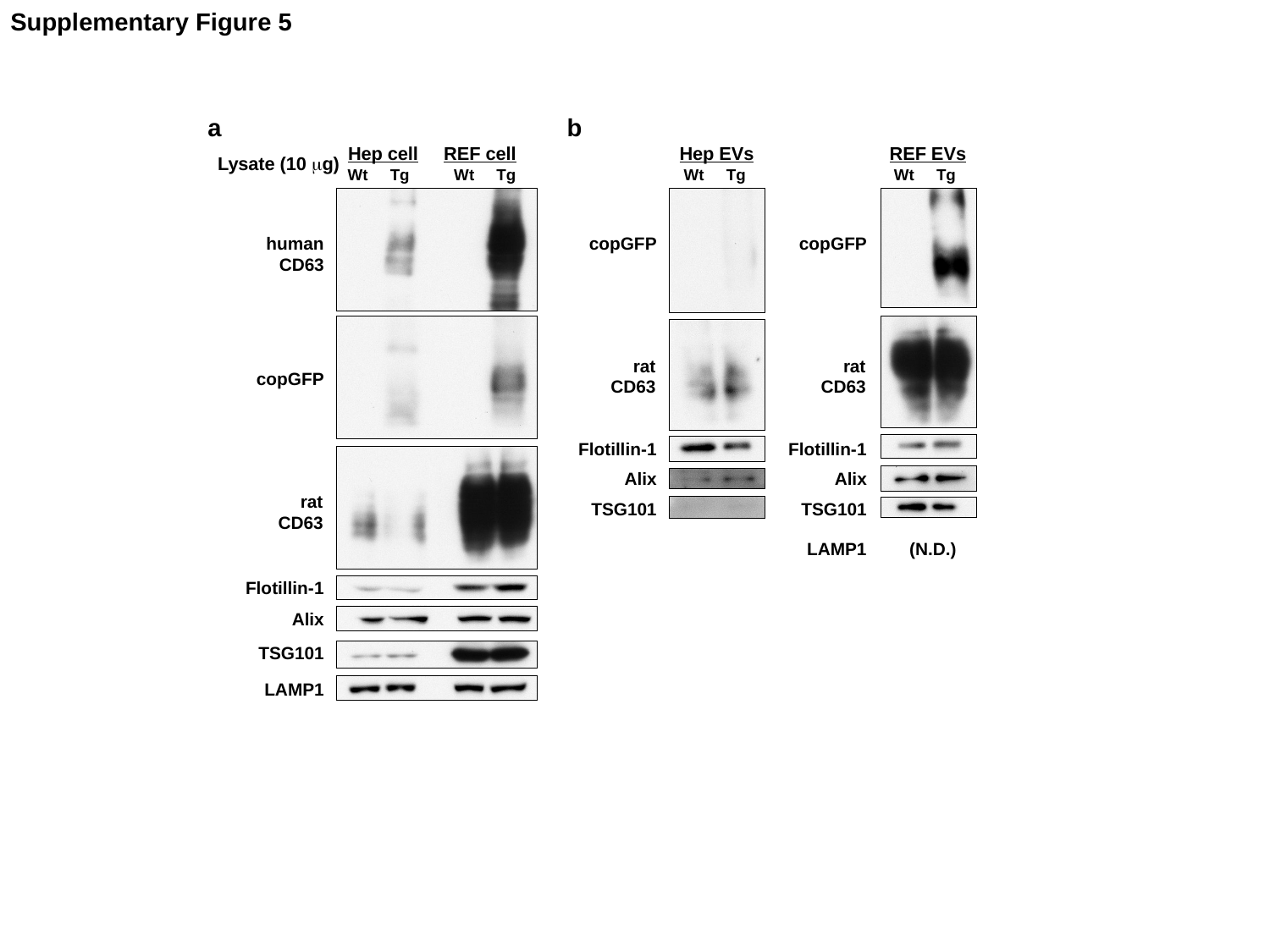

Supplementary Figure 5
a
b
Hep cell REF cell
Wt Tg Wt Tg
Hep EVs
 Wt Tg
copGFP
rat
CD63
Flotillin-1
Alix
TSG101
REF EVs
 Wt Tg
copGFP
rat
CD63
Flotillin-1
Alix
TSG101
LAMP1
(N.D.)
Lysate (10 μg)
human
CD63
copGFP
rat
CD63
Flotillin-1
Alix
TSG101
LAMP1
